# International Dental Federation and International Commission of Education: Joint General Meeting with the American Dental Society of Europe, Stockholm, Sweden, August 16 to 19, 1902

**Published:** 1903-03

**Authors:** 


(To be continued.)

---

### INTERNATIONAL DENTAL FEDERATION AND INTERNATIONAL COMMISSION OF EDUCATION: JOINT GENERAL MEETING WITH THE AMERICAN DENTAL SOCIETY OF EUROPE, STOCKHOLM, SWEDEN, AUGUST 16 TO 19, 1902.

(Continued from page 64.)

*Saturday, August 16.*

THIS session of the Federation was called to order by Professor Lindström.

*Dr. A. W. Harlan.*—MR. PRESIDENT AND GENTLEMEN,—Very unexpectedly I am called upon to say something concerning the object of our meeting here to-day. You will expect from the number who have come from the United States, a long distance, that we are much interested in the object of the International Dental Federation. It seems but a short time ago that a little body of men in Paris gathered together and gave forth the idea that there should be an International Dental Congress. From the moment the idea was broached I cordially and heartily supported it. My *confrères,* many of whom are present to-day, were most earnest in support of the idea of an International Federation, and Professor Brophy and Professor Barrett, and other members interested who are not here to-day, were among the first supporters. Out of that little congress, numbering only a few hundred, at which were present hardly a baker's dozen from the United States, grew the great and successful Columbian Dental Congress in Chicago in 1893. It is not so long ago that any of you will have forgotten the splendid assemblage from countries to the number of twenty-seven who participated in that Congress. After the successful termination of that congress and the publication of its proceedings the congresses were stamped as a success by the profession of the whole world, and when it was intimated, in consequence of the projection of a universal exhibition in Paris in 1900, that another congress was desired, the whole professional dental world to a man gave it its support, and before the termination of that congress the wise men there gathered decided that the interest and enthusiasm excited by the Congress should be further continued by cementing all countries into an International Dental Federation, to concern itself with the teaching of dentistry and make it as nearly uniform as possible.

It was in consequence of that movement in Paris in 1900 that we had the eminently successful meeting in London and Cambridge last year. The success of that meeting was due, I might say, almost wholly to the single efforts of one of our number on the Executive Council, Dr. Cunningham, of Cambridge. Without his invaluable assistance and foresight we would not have had the successful termination of such a large meeting. Its proceedings have been published in all languages and sent everywhere, and to-day, in consequence of the enthusiasm that was spread abroad, the colleges and institutions of learning and societies throughout the world have contributed delegates to be present in this far northern city of Europe, showing by their presence that they are willing to support us, and showing also that they have the one principal central idea, the betterment of the profession and its further universal, international advancement. I thank you for the opportunity of saying these few words, and I hope the deliberations of the Congress will be as successful as have been those of previous years.

*Mr. W. E. Harding* (president of the British Dental Association).—I have very great pleasure in rising to say a few words on behalf of the English dentists and the British Dental Association, of which I have the pleasure and honor of being president this year. That we are very few in number at this international gathering I think must be in a great measure put down to the apprehension that many of my *confrères* have of the inhospitality of the North Sea,—not the inhospitality we should meet here, but that the crossing might be somewhat "lumpy." It is the first occasion on which I have attended a gathering of the International Dental Federation, and, seeing the enthusiasm of the many countries that are represented here, I feel sure that the outcome must be for good. Dentistry, like science, is of no nation, no politics, and no country.

Among the subjects for discussion I see there are several of very great interest. The subject of state dentistry is but now awakening, and we are only on the threshold of what it will be in the not remote future. There are many directions in which the state ought to help us and we the state—if the state will but allow us. I am sure that a gathering like this must help on the good work. Meeting together and rubbing shoulders takes off the angles and points of friction between us, and leads to fraternization which must be of great benefit to our science and our calling. I thank you for the patience with which you have listened to me, and the reception you have given to the English members of this congress, and I hope that the meeting will be for the good of the profession.

*Dr. J. Franck,* Austria.—I have the honor of presenting to you the greetings of the organized body of dentists of Austria and Hungary. Appreciating the importance of the questions on our programme, they have asked me to convey to you their thanks for the disinterested work in which you are engaged for the benefit of the profession at large. Full of hope, they are awaiting the results of this work. I feel happy to be the bearer of these expressions of confraternal feeling from the Austro-Hungarian dentists.

*Dr. Smith Houskin.*—As a delegate of the Norwegian Dental Association I bring a greeting from the Norwegian dentists. We live in a country which to many present may seem to lie far up in the north, away from the great centres of culture, but thanks to the excellent communications of the present day we feel ourselves not so far away after all. Every summer we see in our country great numbers of travellers from south, east, and west many of whom are fond of coming back. But we do not feel ourselves fully satisfied by only seeing visitors in our country from abroad. No! Norwegians from the days of the Vikings to the present moment have had longings for foreign lands themselves. Even as the Vikings of old brought to the country precious treasures,—as for instance the finer culture and Christianity of England, France, and Ireland,—so travel the Norwegians in the present day, and not in the least the Norwegian dentists, to find the wealth and treasure of knowledge and science. From the greater centres there ever blow into our land the fresh winds of a higher culture, and without denying our nationality we take to ourselves all which brings the spirit of man and mankind a step nearer the ideal.

We, therefore, value the international energy of science. For this reason the Norwegian Dental Association looked with interest on the work of the International Dental Federation, and it is the association's great pleasure to get the opportunity of connecting itself with this important society. The pleasure is all the greater as our first meeting with the Federation is here in Stockholm, where we have at the same time an opportunity of meeting so many of our honored Swedish colleagues and friends. We have great expectations of the Federation and its work, for, in the first place, it is by taking up such important questions as dental education and public dental hygiene that we are enabled to reach the greater dental interests, and secondly, because at our head stand men of talent and skill. The energetic president, Dr. Godon, and the untiring secretary, Dr. Sauvez, we all know for their colossal work in connection with the Paris Congress. With the hope that such gentlemen will always stand as the leaders of the Federation, we wish a successful result to the Congress.

*Dr. Weber,* Helsingfors, Finland.—On behalf of the Finnish Dental Society I speak to you now, and I do so with profound feeling. When I think of so large a society with such great men, and between them the little ones, I am overwhelmed. We can all see the great men, but not the little ones; but if for a moment you can observe the little ones, I thank you for that. I am happy that you have recognized me, and further am happy to be the representative of a country from which, though it is not a large one, I bring you greetings. I hold in my hand a document, signed by the vice-president and secretary of the Finnish Dental Society, in which the society presents its greetings and expresses its gratitude for the recognition which has been shown to Finland, and expresses the hope that the Federation will be the means of cementing together the delegates from all countries, including that of Finland.

*Mr. Morrison,* Australia.—I have been called upon equally unexpectedly with others who have spoken before me to address to you a few words. As one of the representatives of an institution which is still in its infancy. I do not think it would be proper to occupy too much of your time, but I may say that the Australian College of Dentistry, although perhaps in its swaddling-clothes, possesses a constitution which is a very robust one. I might also say that the dentists in Australia are doing their best to maintain the dignity of their profession, and to attain a high educational standard. I feel with my co-delegate, Mr. Bain, that our mission here carries with it a great deal of responsibility, but we are amply compensated by knowing that it also bears with it a corresponding high degree of honor. As one of the representatives of a dental institution which is perhaps the leading one of its kind in Australia, I have only to add my thanks for the privilege of being permitted to address to you these few words, and to express a confident assurance that the results of this conference will bear very good fruit in Australia.

*Dr. Guldberg,* Norway.—As president of the Commission of Examinations and as Norwegian delegate on the question of dental education, I have the honor of offering you our respectful greetings and best wishes for the success of this important meeting. Dentistry, as is the case with many other institutions of learning in our country, is a recently organized branch of learning, and thanks to its rapid progress and to the earnest efforts of all interested in this specialty we hope soon to attain our purpose. We are happy to form a part of this distinguished assembly to which so many important men bring their valuable contributions. I beg you, Mr. President, to accept our best wishes for the success of these important meetings.

*Dr. W. E. Royce* (president of the American Dental Society of Europe).—I am pleased to say that the American Dental Society of Europe is very much more ably represented than the delegates which have been appointed to this meeting, but I hope they will not feel I trespass upon their domain if I simply say that that society fully appreciates, possibly more than those who have gained their experience in any one country can appreciate, the importance of the questions which have prompted the promotion of this organization. We most heartily wish for the Federation a long and a successful future.

Addresses were also made by Dr. Frick, of Switzerland, Dr. Artaud, of France, Dr. Guerini, of Italy, and Professor Ehrbehr.

Dr. Sauvez, the secretary-general, read the list of delegates to the Federation meeting.

Dr. Godon then introduced Dr. Brophy, president of the International Commission of Education, to the meeting, and Dr. Brophy took the chair.

### INTERNATIONAL COMMISSION OF EDUCATION.

President Brophy then introduced Professor Lindström, as a man whose name was coextensive with medicine and whose teaching in anatomy was known throughout the world.

*Professor Lindström.*—I have the honor of submitting, at the request of the Swedish delegates to the International Commission of Education, the following short report on the three questions submitted to the consideration of the several delegates by the said Commission:

### REPORT OF DR. LINDSTRÖM, OF SWEDEN, ON THE THREE QUESTIONS PROPOSED.

When the Royal Caroline Institute was intrusted by our government with the organization of dental education in this country, namely, the theoretical and practical studies necessary for the education of dentists, we had to solve to the best of our abilities exactly the same questions as are submitted to our discussion by the International Commission of Education.

The first of these questions is framed as follows: "What should be the preliminary requirements for admission into dental schools? There is only one answer to this question,—viz., that if dentistry is accepted as a true specialty of the healing art, the dental candidate should be required to possess the same preliminary education as that required of the medical student. The answer to the third and last question, which reads, "What part of the studies that are taught in medical schools should be pursued by the dental student?" must be in harmony with the opinion expressed with regard to the first question. It is very plain that the same series of subjects should be studied by the dentist and by the physician, but as the study of medicine in this country requires from nine to ten years, the greater part of which is devoted to the theoretical branches, and as the commission intrusted with the organization of dental education had been asked to limit the length of the course to three years, it became impossible to arrange a plan of studies based upon a common education for both the dentist and the physician.

Following these principles, we organized our dental education five years ago. The curriculum is so arranged that the course is completed in three years. The curriculum embraces a theoretical course during the first year and a practical one during the second and third. Last year some preliminary work on prosthetic technics was introduced in the first year with very satisfactory results. The theoretical course is given by the professors of the medical school, and the practical course is given in a dental clinic especially organized for this purpose and in laboratories of operative dentistry and dental prosthesis, by teachers attached to these institutions.

The theoretical course embraces the following subjects: (1) Chemistry, Metallurgy, and Materia Medica; (2) Anatomy, Histology, and Embryology; (3) Physiology and Medical Physics; (4) General Pathology and Bacteriology.

The subjects of the practical course are as follows: (1) Surgery; (2) Dental Surgery; (3) Obturations; (4) Dental Prosthesis and Orthopædia.

I will now endeavor to answer the other question proposed by the Commission of Education:

*Answer:* The first and second semesters are almost entirely devoted to the study of anatomy. The course is divided into two parts,—dissection and microscopy. The programme comprises general anatomy, systemic and topographic anatomy of the head and neck, special and comparative anatomy of the teeth and mouth; a general survey of the central nervous system and of the organization of the viscera. The course in histology includes general methods of technique, histology of the cells and tissues and special histology of the teeth and neighboring structures. In the course in embryology, a student takes up at the same time the general development of the organic system and the special development of the skull, face, and teeth. I attach a special degree of importance to the evolution of the maxillæ and the teeth after birth, during the period of development, and during senile atrophy. The study of chemistry should comprise the most important features of inorganic and organic chemistry, especially in their relation to dentistry—metallurgy being included in this branch. The study of physiology refers to those parts which have a direct bearing upon the functions of the oral tissues. Materia medica is taken up in so far as it is related to dentistry. Toxicology, antiseptics, and general and local anæsthetics are also included in this group. The course in physiology should comprise the most important points of medical physics and human physiology, especially the physiology of the buccal cavity and neighboring structures. The course in general pathology and bacteriology comprises the most important elements of general pathology and pathological anatomy, the more important phenomena of local changes in the circulation, and passive and active changes of tissues, and must be accompanied by demonstrations by means of anatomical preparations. The course also deals with the most important points in the science of microorganisms and special mycology. After having passed the examinations in the subjects here enumerated, the students follow a course in surgery in the hospital during their second year. This course comprises elements of general surgical pathology, diseases of the buccal cavity and neighboring structures, including diseases of the skin and syphilis and aberrations of development. In Sweden, the students, as a rule, take up the study of dentistry at the age of nineteen or twenty, and after studying three or four years, take the professional examination which confers upon them the right to practise. They enter upon the practice of their profession at an age where we may properly presume that they have a serious conception of their duties as practitioners of dentistry.

*The President.*—Before leaving home I caused the address that I am about to deliver to be printed in French, German, and Swedish. On arriving at Stockholm I found that the Swedish translation was so very poor that it would be misleading, but the French and German are fairly good, though errors are to be found in both.

The report of Dr. Roy, the secretary-general of the International Commission of Education, was read in his absence by Professor Martinier, as follows:

REPORT OF DR. MAURICE ROY ON THE THREE QUESTIONS PROPOSED BY THE INTERNATIONAL COMMISSION OF EDUCATION.

The three propositions which I have the honor to submit to the International Commission of Education of the International Dental Federation in answer to the three questions set forth last year at our London-Cambridge session were first discussed and adopted by the Paris School of Dentistry, afterwards by the commission of education of the National Dental Federation, and lastly by the whole French National Federation at their session of May 25, 1902. I have therefore the advantage of presenting the present report both in my own name and in that of the French National Dental Federation, which represents all the French dental associations, with the exception of two small provincial groups.

With respect to this I take the liberty of drawing your attention to an important point, which is that if, in their deliberations, the International Dental Federation would give, in addition to the personal opinion of their members, that of the national federation, it would afford a larger basis to the discussions of the international assembly. But for that purpose it would be necessary that national dental federations should be organized in every country, which, unfortunately, is not the case. For that reason it is desirable that the Executive Committee of the International Dental Federation should employ all its influence to constitute national federations in all those countries that have representatives among us. It is also desirable that, on their side, these representatives should, each in his respective country, take the initiative in organizations that are so important for the future of the International Dental Federation and for the interests of those whom it proposes to champion.

*First Question.*—"What are the preliminary studies to be required of students before beginning their professional education?"

The necessity of a sufficient general education is admitted by every one, and I think it needless to insist thereon. What we have more particularly to consider is, (1) the extent of such general education; and (2) the age at which it should finish.

These two considerations are, in reality, absolutely inseparable. For if the question of age had not to be considered, there would be no reason why one should limit the general education of the dentist, the general culture of a man being never too great, no matter to what branch of human industry he may be called. But for the dentist a limiting consideration here interposes,—that is, the necessity of his beginning at an early period of life the study of his profession properly so called. This point was made remarkably evident last year at Cambridge by our Philadelphia colleague, Dr. Kirk, and his opinion was supported by the then president, Sir Michael Foster, an eminent physiologist, who summed up in the following short proposition the ideas of our fellow member: "The mind ages slowly and can be educated at even a late period of life, but the body grows quickly old and it is necessary to train it while young."

For these reasons we think that the studies relating to general culture ought not to be prolonged beyond the age of sixteen; not that we think it necessary to commence dental studies at that age, but because we consider that the education of the future dentist should then assume a special character in order to prepare him progressively for dental studies properly so called, beginning thus the formation of the *cone of education* so admirably described by Sir Michael Foster.

What is to be the programme of these studies of general culture? It will evidently be variable according to the country and the educational organization; it ought, nevertheless, to form a complete whole, quite homogeneous, allowing a youth of sixteen to have a general idea of the fundamental bases of human knowledge.

This being recognized as regards general culture, are the studies of the future student of dentistry to be confined solely to this general education, or are they to be already directed to the special object he has to pursue?

In order to solve this question, we must first put another, on the solution of which will depend the answer to the first: What qualifications are necessary to make a dentist? Everybody has not the aptitudes necessary for a dentist, who should possess, besides an intelligence sufficient to assimilate the varied knowledge necessary for the study of our art, enough manual skill to acquire in that time the dexterity required in the execution of the diversified labors of operative dentistry and prosthesis.

Now, there are certain individuals who, though endowed with intelligence in every other respect, are naturally so awkward as to render them incapable of acquiring even medium skill in the practice of our art. It appears, therefore, necessary, according to the principle laid down by Mr. Cunningham, to make sure beforehand that the future student of dentistry possesses this indispensable minimum of manual skill. For this reason it would be advisable to require from the latter a general manual instruction that would enable one to form an opinion as to his aptitude and at the same time would cause him to acquire, from this point of view, the general principles the application of which he would meet with later on. It is also advisable that before entering upon his professional instruction the student of dentistry should possess general scientific notions sufficient to follow profitably the special courses of lectures, or otherwise these would have to be kept at too elementary a level.

It is to these two orders of instruction—gradual manual instruction on the one hand, general and elementary scientific instruction on the other—that the preliminary studies of the student of dentistry must be devoted from sixteen to eighteen, the normal age of admission to the school of dentistry. To these must be added the study of two living languages, the necessity of which has been felt at meetings like these we hold annually.

These different considerations have led us to propose to you to adopt, for the first question put by the International Dental Federation, the following answer, which is the reproduction of a wish expressed by the Congress of 1900:

The *preliminary education necessary* for the student of dentistry before being admitted to follow his professional education must comprise—

(1) A literary education with a knowledge of two living languages.

(2) An elementary knowledge of sciences.

(3) Manual instruction.

On these different orders of instruction we cannot seek for better inspiration than in the remarkable words of Sir Michael Foster when he speaks of the general basis on which all special education must rest. The broad basis, he says, must be "the discipline of the school; that is, the boy's school. I say the discipline rather than the learning of the school, for the arm of the school-master should be in all cases the formation of the mind,—the setting of the instrument, not the filling of the bottle. The growth of habits of accuracy, of intentness, and of alertness, this rather than the gathering of mere knowledge of facts is the proper heritage of the school, and for the attainment of these habits it matters not so much what the boy is taught as how he is taught."

*Second Question.*—"In what are dental studies to consist, how long ought they to last, and what should be the order of the programme?"

This question is incontestably the most important of all that can be put, for it places more particularly at variance the two principles which contend for the direction of dental studies,—the odontologic principle and the stomatologic principle. It is not possible to discuss here these two theories; it has been done too widely to make it necessary to revert to them, and time would be lacking, as the subject is one that admits of long developments. Nevertheless, there are a certain number of points that must be settled, as they form the basis on which we must rely in order to solve the question stated above.

It is incontestable that a man's education is never too extensive; doubtless all knowledge is useful, but, as Sir Michael Foster, in his address at Cambridge, admirably remarks, "The power of the human mind to acquire and preserve knowledge is limited. On the other hand, the development of human knowledge is such that it is no longer possible for a man to be able, like Pic de la Mirandole, to descant *de omni scibili et quibusdam aliis,* and that it becomes indispensable for a man to limit himself if he wants to acquire, not a superficial acquaintance, but a profound knowledge of any branch of science whatsoever. As Dr. Godon remarks, quoting Herbert Spencer, in his excellent address at Cambridge, 'We that have but span-long lives' must ever bear in mind our limited time for acquisition."

The question of how long studies ought to last is therefore of extreme importance, as argued last year by Dr. Godon and by myself in the discussions of the International Federation. Their duration must be perforce limited, and four or five years seems to be a maximum that cannot be exceeded. It would be impossible to require from dentists studies analogous to those of doctors of medicine, since it is impossible, as no one sufficiently acquainted with the facts will deny, in four or five years to make at once a doctor and a dentist. It is advisable therefore to make a rational use of the maximum time to be devoted to study, so as to allow of students of dentistry acquiring gradually the knowledge requisite for the exercise of their profession.

Dental studies come under two heads: on the one hand scientific and medical studies, on the other technical studies. How are they to be divided?

We consider it indispensable to give a very notable pre-eminence to technical studies, and particularly to practical work. Dental therapeutics and its two corollaries, operative dentistry and prosthesis, admit of a very important manual part, necessitating a long apprenticeship both of the eye and of the hand, hence the necessity of devoting a considerable part of the hours allotted to study to practical work (prosthesis and operative dentistry).

According to the calculations made by Dr. Godon in his thesis and previously by myself in my report to the Congress of 1900, a proportion of one-fifth of the hours of study devoted to the scientific and medical part of dental instruction appears to be sufficient. We think, therefore, that this proportion may be accepted as a basis on which the programme of this part of dental instruction should be established.

Is it desirable to divide the teaching as is done in certain foreign countries—in Switzerland, for instance, where one year is devoted to the study of physical and natural sciences, one year to medical studies, and a year and a half to dental studies? We are not of that opinion. We have already said that technical studies should be of long duration, particularly as regards practical work; now, in order that the student may profit by the latter, it is necessary that he should receive concurrently theoretical technical instruction without prejudice to general scientific and medical instruction. From the above facts the necessity follows of organizing different courses of lectures, so that medical and scientific instruction and technical instruction may be given simultaneouslv. The distribution alone should differ; scientific and medical lectures should predominate in the first years and technical lectures in the last. All the courses of lectures, especially the technical ones, should naturally be graduated according to the years of study.

It is desirable that a particular regulation should be made applying to doctors of medicine who intend to practise dentistry; and, conformably to the wish expressed by the Congress of 1900, it should be obligatory for this category of students to attend for two years the classes of a school of dentistry. It would, however, be necessary to draw up a programme of practical work for these students so as to allow of their going gradually through the programme of practical work which normally extends over four years.

These diverse considerations being settled, we propose to you to adopt the following answer to the second question above stated:

(1) Dental studies comprise a scientific and medical part, and a technical part.

(2) Their duration must be at least four years.

(3) These studies must be organized conformably to a parallel method for all the classes—scientific and medical instruction and technical instruction simultaneously.

(4) It must be obligatory on medical graduates who purpose practising the art of dentistry to attend, for at least two years, the lectures of a school of dentistry.

*Third Question.*—" What branch of the studies, as taught in medical schools, must the student of dentistry follow?"

After what we have just said on the preceding question, the answer to this last question does not admit of being extended. In the first place we reject completely, for reasons that have been often stated and of which a few are here recalled, the assimilation of dental studies to medical studies and consequently the obligation of complete medical studies in order to practise the art of dentistry.

Irrespective of this absolute view of the question, is there an advantage for students of dentistry to follow certain teachings given in schools of medicine? We think not. As we have already said in our report on instruction, if it be indispensable that the student of dentistry should have a knowledge of anatomy, physiology, chemistry, and physics, it is not indispensable that his knowledge of these subjects should be as extensive as if he were destined to practise medicine or to take a degree in physical sciences. In the faculties of medicine lectures are given with a view to medical studies; it follows, therefore, that students of dentistry, having but a limited time to devote to them, can profit only to a limited extent by lectures of a higher order than is strictly necessary for their later studies, or else, should they devote sufficient time to them, unless their studies be prolonged it must be to the detriment of their professional instruction.

I am happy to find that on this point my opinion coincides with that of a man of intellect, Dr. Joseph Griffiths, professor of surgery at the University of Cambridge. In the memorable session of the International Dental Federation at Cambridge Dr. Joseph Griffiths, after having compared the respective wants of the doctor and the dentist, said, in his remarkable discourse, "Can this education of a dentist be carried on side by side with that of the medical men? is the question of practical importance. I would unhesitatingly answer, No. The anatomist may train either, but he cannot train both together without giving one much more than he requires and not paying enough attention to the other. It is much the same with physiology. Therefore, I say their courses should be separate, and so arranged as to serve the right end."

The conclusion we arrive at is that if dental science is an autonomous science, then dental instruction must be autonomous instruction.

There remain, however, two particular points to be examined: dissection on the one hand, medical and surgical clinics on the other.

As regards dissection, dental schools have a difficulty to contend with, —namely, that of procuring corpses, many considerations interposing in many countries with respect to this. In countries where this difficulty exists it is absolutely necessary to have recourse to medical schools or to hospitals, but subject to one peremptory condition,—namely, that lectures shall be given there exclusively for the use of dentists, the *locale* alone being changed, the spirit of the teaching remaining the same.

With respect to medical and surgical clinics the question is easier to solve. We must bear in mind that with very rare exceptions—and then they are generally assisted by the doctor in attendance—dentists have only to attend patients who are not confined to their beds. Consequently what they have an interest in seeing is patients coming and going like those who present themselves at the consultations for out-door patients. It is therefore easy to have a consultation room annexed to each school, in order to supply this want; but, in default of such a plan, it will be necessary to apply to the hospitals, bearing in mind, as for dissection, that the examination of patients be made strictly with a view to the special teaching to be followed by students of dentistry.

We therefore propose to you, in answer to the third question, to adopt the following resolution:

"All the studies of students of dentistry must take place exclusively in schools of dentistry, except that of dissection in cases where it is impossible to procure dead bodies, but subject to the condition that the lectures be given exclusively for the use of such students."

Such are the reasons which have led us to adopt the three resolutions which I have the honor to submit for the approval of the International

Dental Federation in the name of the French National Dental Federation, which resolutions I reproduce below:

First resolution:

The preliminary education necessary for the student of dentistry before being admitted to follow his professional education must comprise—

(1) A literary education with a knowledge of two living languages.

(2) An elementary knowledge of sciences.

(3) Manual instruction.

Second resolution:

(1) Dental studies comprise a scientific and medical part and a technical part.

(2) Their duration is at least four years.

(3) These studies must be organized conformably to a parallel method for all the classes: scientific and medical instruction and technical instruction simultaneously.

(4) It must be obligatory on medical graduates who purpose practising the art of dentistry to attend, for at least two years, the lectures of a school of dentistry.

Third resolution:

All the studies of students of dentistry must take place exclusively in schools of dentistry, except that of dissection in cases where it is impossible to procure dead bodies, but subject to the condition that the lectures be given exclusively for the use of such students.

Dr. Bogue wished to know whether the report had been printed in English?

Dr. Godon said that the committee by great efforts had had the reports put into English and printed, at some expense, and a copy had been sent to every member. There were very few remaining, and it was impossible to distribute them to all present.

Dr. Holly Smith wished to have a discussion on Dr. Brophy's paper without any extraneous matter being introduced.

The President (Dr. Brophy) said he could clear up the whole matter by stating that the report read by the Secretary was in English in his hands, and it could be read in English if necessary.

